# Major vacuolar TPC1 channel in stress signaling: what matters, K^+^, Ca^2+^ conductance or an ion-flux independent mechanism?

**DOI:** 10.1007/s44154-022-00055-0

**Published:** 2022-08-11

**Authors:** Igor Pottosin, Oxana Dobrovinskaya

**Affiliations:** 1 Biomedical Center, University of Colima, 28045 Colima, Mexico; 2 International Research Centre for Environmental Membrane Biology, Foshan University, Foshan, 528041 China

**Keywords:** TPC1, Potassium, Calcium, Voltage gating, Long-distance signaling, Vacuole

## Abstract

Two-pore cation channel, TPC1, is ubiquitous in the vacuolar membrane of terrestrial plants and mediates the long distance signaling upon biotic and abiotic stresses. It possesses a wide pore, which transports small mono- and divalent cations. K^+^ is transported more than 10-fold faster than Ca^2+^, which binds with a higher affinity within the pore. Key pore residues, responsible for Ca^2+^ binding, have been recently identified. There is also a substantial progress in the mechanistic and structural understanding of the plant TPC1 gating by membrane voltage and cytosolic and luminal Ca^2+^. Collectively, these gating factors at resting conditions strongly reduce the potentially lethal Ca^2+^ leak from the vacuole. Such tight control is impressive, bearing in mind high unitary conductance of the TPC1 and its abundance, with thousands of active channel copies per vacuole. But it remains a mystery how this high threshold is overcome upon signaling, and what type of signal is emitted by TPC1, whether it is Ca^2+^ or electrical one, or a transduction via protein conformational change, independent on ion conductance. Here we discuss non-exclusive scenarios for the TPC1 integration into Ca^2+^, ROS and electrical signaling.

## Origin and distribution of TPC1

**Fig. 1:**
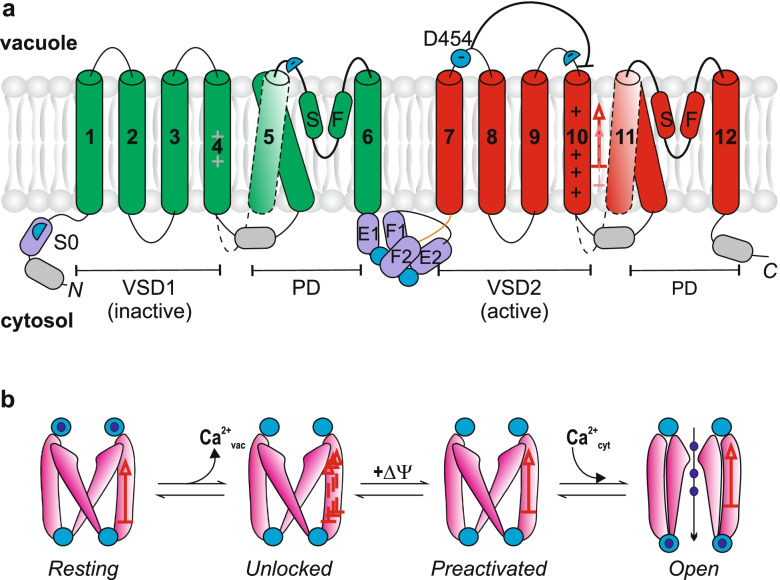
Topology and gating scheme for the plant TPC1. **a** Plant TPC1 consists of the two 6-TM domains (membrane helices are numbered from 1 to 12). Each 6-TM domain can be divided into voltage-sensing (VSD) and pore domains (PD). SF is the selectivity filter. E1,2 and F1,2 are parts of cytosolic Ca^2+^ − binding EF-hands. Two negative residues (Asp) in S0 likely form a part of the Ca^2+^-coordination site together with EF2; Ca^2+^ binding of this site is transmitted to the dilation of the cytosolic mouth and pore opening, providing the VSD2 is in the upper position. Critical residues, responsible for the channel inhibition by vacuolar Ca^2+^ are shown in blue; D454 is an Asp residue, whose mutation is responsible for the *fou2* mutation, insensitive to luminal Ca^2+^. **b** A minimal scheme, depicting the sequence of events, leading to the TPC1 opening. At the first step the luminal Ca^2+^ ions are removed, unleashing the VSD2 movements. Application of cytosol positive voltage stabilizes the VSD2 in the upper position; in this configuration the channel is able to bind cytosolic Ca^2+^. Finally, binding of cytosolic Ca^2+^ promotes the channel opening

TPC1, a Two-Pore Cation 1 channel, is widespread in animal and plant kingdoms. It is targeted to the membranes, delineating acidic intracellular compartments, vacuoles in plants and lysosomes in animals. TPC1 is a product of a duplication of the six transmembrane domain (6-TM) Shaker potassium channel gene, whereas a further duplication generated voltage-dependent Na^+^ and Ca^2+^ channels, which are ubiquitous in animals, but absent in higher plants (Anschütz et al. 2014). Animal (hTPC1) and plant (a prototypic AtTPC1) are different in the nature of the gating process. The first one is activated by a ligand, whereas plant AtTPC1 possess several canonical Ca^2+^-binding sites (EF-hands) and are activated by cytosolic Ca^2+^ (Fig. 1a). Both hTPC1 and AtTPC1 are activated by a depolarization (Cang et al. 2014; Hedrich et al. 2018). Originally, it was assumed that, being both Ca^2+^-permeable, the animal TPC1 should act as a generator/trigger of Ca^2+^ signal, whereas plant ATPC1 should act as an amplifier of the initial Ca^2+^ stimulus (Patel et al. 2011). Study on isolated vacuoles showed that the vacuolar Ca^2+^ release shares the pharmacological profile of TPC1, which appears to be the only Ca^2+^-permeable channel of the tonoplast (Pottosin et al. 2009). Later, it was shown that hTPC1 actually forms a Na^+^-selective channel with a very limited Ca^2+^ and K^+^ permeability (Cang et al. 2014). When it comes to plant AtTPC1, the physiological importance of its Ca^2+^ permeability is currently a matter of discussion.

TPC1 can be found in some Charophyte algae and in all terrestrial plants. Recent phylogenetic study revealed two distinct clades of TPC1 in terrestrial plants. Clade *a* includes TPC1 from angiosperms, ferns and bryophytes. It is characterized by highly conserved binding sites for cytosolic and vacuolar Ca^2+^, which are not conserved in the clade *b* (mosses and liverworts) (Dreyer et al. 2021)*.*

## TPC1 is involved in plant responses to stress

Despite its ubiquitous and abundant expression in the tonoplast, the functional role of the TPC1 channels in plants for many years remained cryptic. Recent studies demonstrated its involvement in different stress responses (Table 1). Of note, the experimental evidence just says that the TPC1 expression is essential for a stress response, but not specifies the way how it is achieved. It is very tempting to link the involvement of an ion channel into a physiological response via its ion-conducting activity and, in most cases, it turns to be true. Yet, when it comes to TPC1, this has not been yet proved experimentally, so that the authors of studies mentioned in the Table 1 generally avoided to conclude whether the intervention of the TPC1 was direct or indirect.

**Table 1:** Expression of TPC1 affects plant responses to stressors

Stress stimulus	Affected process	Experimental evidence	Proposed mechanism	Reference
High external Ca^2+^	Stomata closure	Loss-of-function *attpc1–2* mutation impairs stomata closure induced by high Ca^2+^ but not by ABA or methyl jasmonate	Ca^2+^-dependent priming of the plasma membrane S-type anion channel activity, independent on oscillations of global Ca^2+^	Peiter et al.*, *2005 Islam et al., 2010
Local high NaCl	Long distance Ca^2+^ and ROS signaling along the root	Loss-of-function *attpc1–2* mutation strongly decelerates and TPC1 overexpression accelerates the Ca^2+^ wave	Crosstalk with a ROS-activated Ca^2+^ influx channel of plasma membrane, involves the activity of NADPH-oxidase. Fire-diffuse-fire vacuolar Ca^2+^ release model, which requires the presence of TPC1 (not necessarily as a direct Ca^2+^ source)	Choi et al., 2014 Evans et al., 2016
Mechanical wounding, herbivory attack	Systemic Ca^2+^ signal between treated and adjacent leaves through a direct vascular connection	Loss-of-function *attpc1–2* mutation does not affect local but impedes systemic Ca^2+^ increase	TPC1 is neither involved in the generation of the local Ca^2+^ signal nor in the transmission (likely, via the electropotential link, mediated by GLR channels activity in phloem, which lacks vacuoles). Only acting downstream in the target leaf cells (?)	Kiep et al., 2015
Aphid feeding	Intracellular Ca^2+^ rise around aphid feeding site	*attpc1–2* mutation reduces Ca^2+^ rise around aphid feeding site, hyperactive TPC1 *fou2* mutation assists systemic Ca^2+^ response	Dependent on vacuolar TPC1 and plasma membrane GLR3.3 and GLR3.6. Believed to be a summation of Ca^2+^ influx via GLRs and vacuolar Ca^2+^ release, directly or indirectly controlled by TPC1	Vincent et al., 2017
Electrical stimulation of the tonoplast	Tonoplast excitability. Generation of the post-stimulus tonoplast depolarization plateau	The prolonged depolarization is impaired in *attpc1–2* and upon TPC1 inhibition by luminal Ca^2+^; the depolarization becomes permanent in *fou2* insensitive to luminal Ca^2+^	Tonoplast depolarization is provoked by vacuolar K^+^ release and is dependent also on the activity of vacuolar K^+^ selective channels TPK. TPKs make a direct contribution, whereas TPC1 activation is transmitted somehow to that of TPKs	Jaślan et al., 2019

## Selectivity and conductance: a relation between fractional K^+^ and Ca^2+^ fluxes

TPC1 channels in plants are almost perfectly charge-selective, i.e. they exclude anions but conduct small monovalent and divalent cations almost indiscriminately. Divalent cations such as Mg^2+^, Ca^2+^ and Ba^2+^ bind much stronger within the pore than monovalent ones. Thus, whereas Na^+^ and K^+^ conductance of TPC1 channels can reach impressive 200 pS at physiologically attainable concentrations for these cations, the limiting conductance for stronger bound, hence, slower passing, Ca^2+^ and Mg^2+^ is 13 and 18 pS, respectively (Pottosin et al. 2001; Pottosin and Dobrovinskaya 2014). Typical vacuole contains 0.2–2.0 mM of free Ca^2+^, and Mg^2+^ is in the millimolar range, whereas vacuolar K^+^ under non-stressed conditions ranges between 100 and 200 mM (450 mM in an open stomata) and Na^+^ can be accumulated to even higher concentrations under a severe salt stress. On the background of 100 mM K^+^ in the vacuole, 2 mM vacuolar Ca^2+^ or Mg^2+^ approximately twice reduced the TPC1 current, mainly mediated by K^+^ (Pottosin and Dobrovinskaya 2014), which implies that, despite the prevalence of K^+^ in the lumen, the TPC1 pore is roughly halftime occupied by Ca^2+^ or Mg^2+^. The determination of the fractional K^+^ and Ca^2+^ fluxes is not a trivial task, because, apart from a competition between two cations for the pore occupancy, the magnitude and direction of the ion flux depends also on the driving force, determined by the electrochemical potential difference for a given cation. A more than 1000-fold cytosol directed gradient of free Ca^2+^ implies that TPC1 can only mediate the vacuolar Ca^2+^ release, basing on the estimated E_Ca_ ~ + 100 mV and transtonoplast electrical potential difference of − 30- 0 mV. Parallel measurement at variable membrane potentials of a net (mainly K^+^) TPC1-mediated current by patch-clamp and Ca^2+^ flux by a fluorescent dye yielded the fractional Ca^2+^ current of 5–10% of the total one (Gradogna et al. 2009). This estimate was only possible at highly unphysiological conditions, with 0.5–2.0 mM cytosolic Ca^2+^, to ensure the full activation of TPC1 channels, so that measured Ca^2+^ fluxes reflected Ca^2+^ uptake by the vacuole. Usage of a more sensitive MIFE technique to measure tiny cytosol-directed Ca^2+^ fluxes from intact vacuoles at ionic conditions approaching physiological ones and parallel measurements of the TPC1 activity by patch-clamp yielded a fractional Ca^2+^ current of ~ 0.1 pA per single TPC1 at resting vacuolar potentials, as compared to TPC1-mediated single channel K^+^ current, which is in low pA range (Pérez et al. 2008). It may be concluded then that fractional Ca^2+^ is about or less than 10% of the total current at physiological conditions. Therefore, Ca^2+^ flux contribution to the tonoplast polarization can be neglected, but not yet its potential role in Ca^2+^ signaling.

The fact that the TPC1 channel can mediate Mg^2+^ current in the pA range forced us to conclude that Mg^2+^ passed the narrowest pore constriction in a fully hydrated form, because if its transport of Mg^2+^ should be limited by a dehydration respective current will be in the fA range (Pottosin and Schönknecht 2007). Indeed, the structural study revealed that the narrowest part of the plant TPC1 channel pore is short and wide (Guo et al. 2016) which makes the transport of hydrated cations feasible, in contrast to hTPC1, where Arg residues at the external end of the pore constriction (part of selectivity filter, SF, shared by a 2nd 6-TM domain) reduced the cutoff limit to 4.8 Å and therefore makes the channel Na^+^-selective (Guo et al. 2017). At this point it is important to note that the nature of SF in an TPC1 channel and a Shaker K^+^-selective channel is different. Long and narrow SF in K^+^-selective channels contains 4 K^+^ binding sites, of which two should be occupied simultaneously to allow fast K^+^ transport due to a mutual electrostatic repelling between neighboring K^+^ ions (Zhou et al. 2001). SF (taken as the narrowest pore constriction) in the TPC1 channel contains no binding sites for divalent cations (Guo et al. 2016). When Ba^2+^ ions were used as Ca^2+^ homologues, the electronic density study revealed three preferable Ba^2+^ localizations: two in the large water-filled cavity beneath the SF and one above the SF at the external (luminal) pore entry. Recent in silico study specified two residues in the extracytosolic part of the AtTPC1 pore, just next to the SF, whose mutation to Ala primarily affected (handicapped) Ca^2+^ conductance but to a much lesser extent affected the conductance of monovalent cations (Navarro-Retamal et al. 2021). Our independent docking study (Pottosin et al. 2021) revealed that the same two residues, which control the Ca^2+^ permeation, are crucial for binding of long polyamines, PAs (like spermine, Spm); these residues do not participate in the binding of a short diamine putrescine, Put. PAs are natural blockers of the TPC1 channels. The concentration of PAs is differentially modulated by stresses. For instance, K^+^ deficiency is associated with a high Put level, whereas salt stress is normally associated with a high Spm (Pottosin and Shabala 2014; Cui et al. 2020). One may presume that the mutation of aforementioned key residues to Ala will result in a phenotype less sensitive to Spm (but not to Put) and a higher preference for monovalent cations, Na^+^ and K^+^ over Ca^2+^. These may imply a different (supposedly, higher) sensitivity of the mutants to high salinity. And, using these mutants, one can elucidate whether Ca^2+^ conductance of the TPC1 is physiologically important. Recent cryo-EM structural study revealed additional residues, which coordinated the external (luminal) cation-binding site, although it could not be discerned whether observed bound cation was Ca^2+^ or Na^+^ due to their very similar diameter (Dickinson et al. 2022). The last study revealed also an unexpected result that the mutation of some of these residues affected also the TPC1 channel gating by membrane voltage and luminal Ca^2+^.

## How TPC1 senses the transmembrane electrochemical gradients for K^+^ and Ca^2+^

Typical vacuole, being the main Ca^2+^ store in a plant cell, usually contains several thousand active TPC1 copies (Schulz-Lessdorf and Hedrich 1995; Pérez et al. 2008). A simultaneous opening of just few TPC1 channels will dissipate the electrochemical gradients for ions across the tonoplast, whereas global vacuolar Ca^2+^ release will oversaturate the cytosol with a lethal Ca^2+^ concentration. To avoid this scenario, a high threshold is set for the TPC1 channels opening. First, the TPC1 is gated on by unphysiological cytosol positive voltages. Another factor, which increases the voltage threshold for the TPC1 opening to even higher positive potentials is the luminal Ca^2+^. The detailed kinetic model for the TPC1 regulation by luminal Ca^2+^ and Mg^2+^ is available (Pottosin et al. 2004; Pottosin and Dobrovinskaya 2016). The stabilization of the channel resting state is due to much stronger (apparent K_d_ value of few μM as compared to 0.1–10 mM range for vacuolar Ca^2+^ concentration) luminal Ca^2+^ binding as compared to its affinity in the intermediate and open channel conformations; Mg^2+^ binding was by more than an order of magnitude weaker (Pottosin et al. 2004). TPC1 channels are activated by cytosolic Ca^2+^ and may not be open even at very high positive voltages in Ca^2+^ free solutions. We have observed only brief, about 10 ms, stochastic TPC1 openings at these conditions, one opening per several minutes of recording or an open probability less than 10^− 4^. The fact that TPC1 is activated by cytosolic Ca^2+^ was taken as glimpse that it can mediate Ca^2+^-induced Ca^2+^ release (CICR), in analogy to CICR mediated by ryanodine receptor channels of the endoplasmic reticulum in animal cells (Ward and Schroeder 1994). Such a global involvement is of course impossible for a vacuolar Ca^2+^-permeable channel, as far as vacuole represents a non-exhaustive Ca^2+^ store. TPC1 indeed did not significantly contribute to global Ca^2+^ signaling in a plant cell (Ranf et al. 2008). It should be noted here that the apparent K_d_ value for TPC1 activation by cytosolic Ca^2+^ is in the submillimolar range (Schulze et al. 2011; Guo et al. 2016; Demidchik et al. 2018), whereas bulk free cytosolic Ca^2+^ can reach 1–2 μM at the best upon signaling responses (Ranf et al. 2008). Overall, the TPC1 senses luminal and cytosolic Ca^2+^ as if its activity is controlled by electrochemical gradient for this ion across the tonoplast. The relation is reciprocal: the channel is gated open by Ca^2+^ gradient opposite to physiological one, whereas at physiological vacuole-to-cytosol directed Ca^2+^ gradient the ion leak via TPC1 channels is strongly reduced (Pottosin et al. 1997). For isolated vacuoles at resting values of cytosolic Ca^2+^ there is just one out of several thousand active copies of the TPC1 in a vacuole open at time and about ten at very high (20 μM) cytosolic Ca^2+^ (Pérez et al. 2008; Pottosin and Dobrovinskaya 2016). Luminal cations other than Ca^2+^ affected the voltage dependence of the TPC1. In case of K^+^ and Na^+^ the voltage dependence shifts to more positive potentials at higher cation concentration, so that the higher is the driving force for the vacuolar cation leak the higher will be the threshold for the channel voltage activation (Pottosin et al. 2005; Pérez et al. 2008). For K^+^ this effect is a consequence of a non-specific shielding of the negative charge at the membrane surface so that at the same nominal voltage difference between bulk phases the intermembrane portion of the cytosol minus vacuole voltage drop becomes less positive (Pottosin et al. 2005). The effect of Na^+^ is qualitatively similar but quantitatively stronger than that of the equimolar concentration of K^+^ (Pérez et al. 2008). The effects of increased Ca^2+^, Na^+^ and K^+^ cytosol-directed gradients on the channel voltage dependence are not additive. Rather, increased luminal K^+^ and Na^+^ concentration desensitized the channel towards luminal Ca^2+^ binding (Pottosin et al. 2005; Pérez et al. 2008). In addition, protonation of the luminal Ca^2+^ binding sites at physiological vacuolar pH (5.5) decreased their affinity to Ca^2+^ (Pérez et al. 2008; Pottosin and Dobrovinskaya 2016). Specifically, the combined effect of the vacuolar cation mixture makes TPC1 activity almost insensitive to vacuolar Ca^2+^ variation within its physiological range of concentrations, 0.1–1 mM, yet preserving a strong dependence of voltage threshold on luminal Ca^2+^ both at lower and higher concentrations (Pérez et al. 2008). Before considering the details of conformational changes, associated with the TPC1 gating by luminal and cytosolic Ca^2+^ as well as by voltage, it is worth to mention that nowadays we understand quite well how the activity of TPC1 channels is restricted to an absolute minimum under resting conditions, principally, physiological voltage and Ca^2+^ gradient. What we do not understand yet is how these apparently silent channels are recruited into the signaling processes. The response to this question can be non-trivial. A long search for auxiliary intracellular factors, which may decrease the threshold for the TPC1 activation so far was unsuccessful (reviewed by Pottosin and Dobrovinskaya 2016). Thus, here we will not consider the intervention of these not yet identified factors.

## Structure-functional relations for the TPC1 gating by Ca^2+^ and voltage

TPC1 is mainly in the closed resting state at physiological tonoplast potentials, which range from zero to − 30 mV. In contrast to this, most of voltage-dependent Shaker and Shaker-derived channels are already open at zero voltage. Thus, in structural studies of membrane-free protein complexes such channels but TPC1 are captured in the open state conformation. Shaker K^+^ and Shaker-derived cation channels possess the voltage sensing domain, VSD, which is comprised of 4 helices, one of which (S4) bears several positively charged residues (Fig. 1a). For AtTPC1 the S4 in the VSD1 is immobile, hence only VSD2 is active in voltage sensing and transduction (Guo et al. 2016; Jaślan et al. 2016). Wild type TPC1 is crystalized or frozen in the resting state and respective structure is available at high resolution (Guo et al. 2016; Dickinson et al. 2022). To reveal the structural changes occurring in steps from the resting state towards opening, the hyperactive mutants insensitive to luminal Ca^2+^ have been employed. In AtTPC1 the mutation of a single Asp454 at the luminal face of the protein abolished the sensitivity to vacuolar Ca^2+^ (Beyhl et al. 2009; Dadacz-Norloch et al. 2011). This mutant, called *fou2*, displays a hyperactive AtTPC1 and increased production of biotic stress hormone jasmonate, resulting in a higher resistance to fungal or herbivore attack (Bonaventura et al. 2007). Usage of *fou2* or a mutant in which also two other luminal Ca^2+^ binding residues, Asp240 and Glu528 (Fig. 1a), were mutated to alanine, allowed to obtain the structure of the intermediate conformational state(s), yet not open, but primed for opening. Notably, obtained structures were similar to that of the wild type channel at Ca^2+^-free conditions (Ye et al. 2021; Dickinson et al. 2022). But the advantage of such mutants was the possibility to generate fully open state by increasing Ca^2+^ concentration in the medium. The removing of the inhibitory Ca^2+^ binding site unfastened the VSD movement. The transition from the resting to primed state likely occurs in two steps characterized by relatively large conformational changes in the VSD2: S7-S9 moves rotationally around the S10 and S10 moves laterally and “upwards” (towards the cytosolic interface) (Dickinson et al. 2022). The multistep voltage dependent process towards the channel opening is consistent with a delay in the kinetics of the TPC1 activation in a response to instantaneous depolarization and a biphasic dependence of a steady state activity on membrane voltage, which implies multiple closed states, while transitions between them and the open state have a different voltage sensitivity. Closed states and open state display a different affinity to luminal Ca^2+^, which progressively decreases many-fold while processing from the distal closed (resting) state to the open one (Pottosin et al. 2004). The final state becomes competent to cytosolic Ca^2+^ binding, which is a pre-requisite for the eventual channel opening. The movement of the VSD2 allowed a global conformational change in the cytosolic Ca^2+^-binding domain EF2, which consists of a local intrinsic movement and global rotation. This deblocked two Ca^2+^ binding sites, one in EF2 and another in S0; binding of Ca^2+^ to these sites causes the opening of the cytosolic gate of the channel pore, likely via a direct mechanical transduction from S0 to the pore helix S6 (Ye et al. 2021). The recently identified additional Ca^2+^ binding site is coordinated by side chains of Asp39 and Asp43; their mutation greatly reduced the fraction of the TPC1 channels, which can be activated by cytosolic Ca^2+^ and voltage (Ye et al. 2021). Functional studies suggest the presence of at least two types of cytosolic Ca^2+^ binding sites. The receptor site is specific for Ca^2+^ so that only Ca^2+^ binding to it turns the channel on. In the modulatory site Ca^2+^ can be replaced by Mg^2+^; both cations sensitize the channel towards Ca^2+^ binding at the receptor site (Pei et al. 1999; Carpaneto et al. 2001; Pérez et al. 2008; Schulze et al. 2011). Ca^2+^ binding to the receptor site is associated with the increase of fraction of TPC1 channels open at high positive potentials; cooperative interaction between receptor and modulatory sites is manifested by the negative shift of the voltage dependence upon cytosolic Ca^2+^ increase (Schulze et al. 2011; Demidchik et al. 2018). As TPC1 possesses multiple cytosolic Ca^2+^ binding sites, their definite attribution to receptor and modulatory ones is not possible for the moment, but some preliminary conclusions can be made: EF1 in the first 6TM domain of the TPC1 is not the receptor site, but can be part of the modulatory one, whereas EF2 together with S0 is a part of the receptor site (Schulze et al. 2011; Ye et al. 2021). For the understanding of the next section it is important to stress that TPC1 opening occurs in multiple steps (Fig. 1b) and respective conformational states are structurally different, which can be eventually sensed.

### TPC1: waking up a sleeping giant

The situation with the TPC1 is like a story of a bumblebee: the theory predicts it cannot fly, but it flies! Almost all TPC1 activity measurements were done at experimental conditions favoring the channel opening. These typically are zero vacuolar Ca^2+^, high (at least, 10 μM) cytosolic Ca^2+^ and high positive voltages, all unphysiological. Such experiments demonstrate enormous potential of the TPC1 channels, which are capable to generate huge (nA) currents per vacuole. At the same time, a rather sensitive non-invasive MIFE technique is required instead of patch-clamp to measure tiny ion fluxes, when it comes to physiological conditions. Yet, even at elevated (2 μM) cytosolic Ca^2+^ the cytosol directed net Ca^2+^ flux was equivalent to just 1.5 pA per vacuole (Pérez et al. 2008). But, on the other hand, there is plenty of recent evidence that functional TPC1 channels are required to speed up Ca^2+^ wave induced by a local high NaCl application to roots (Choi et al. 2014; Evans et al. 2016), for Ca^2+^-dependent stomata closure (Islam et al., 2010) and systemic Ca^2+^ signaling upon herbivore attack and aphid feeding (Kiep et al. 20,215; Vincent et al. 2017). There is no doubt that TPC1 channels work, but we do not know exactly how. Let us consider different non-exclusive possibilities.

#### TPC1 channels directly release Ca^2+^ from the vacuole

**Fig. 2:**
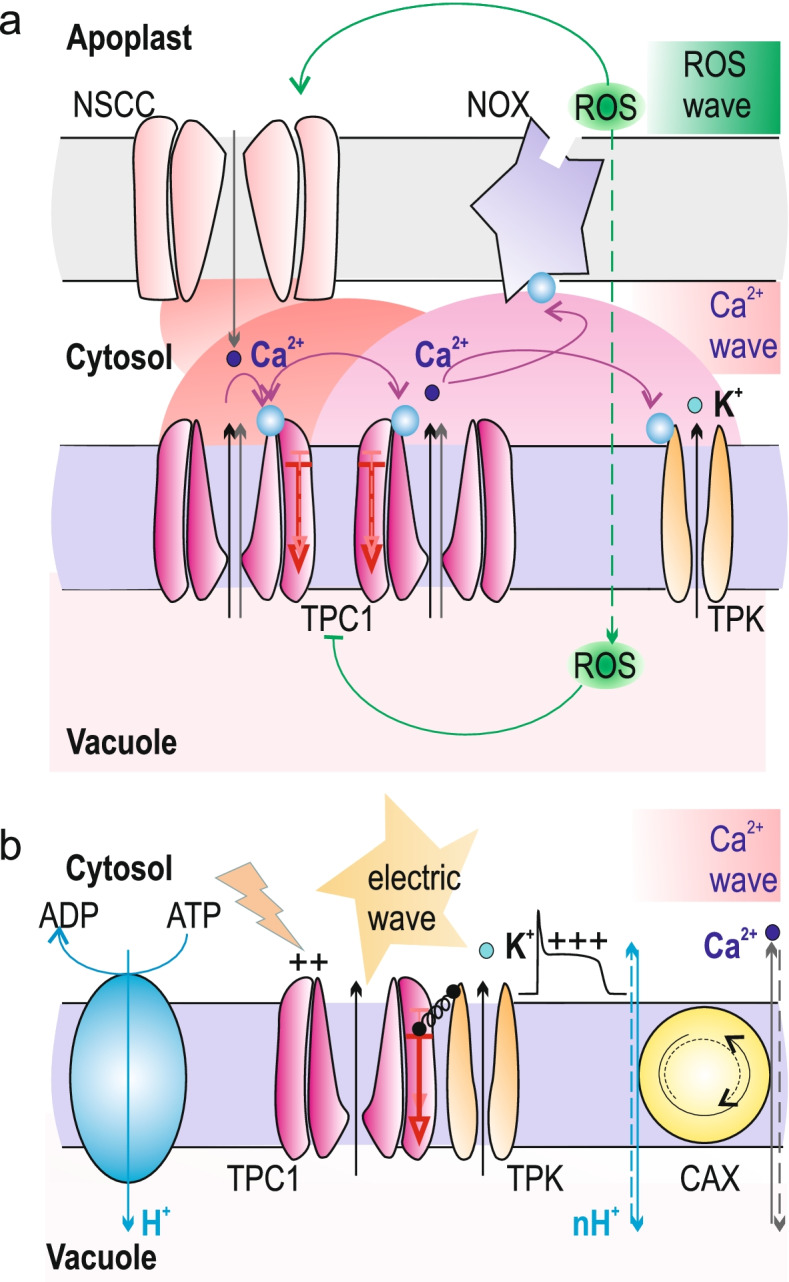
Hypothetical non-exclusive mechanisms for the TPC1 integration in signaling processes. **a** TPC1 and Ca^2+^ and ROS signaling. Ca^2+^-activated ROS producing NOX and ROS-activated Ca^2+^-permeable NSCC (which can be GlR 3.3/3.6) in the plasma membrane, PM, form a self-amplifying loop. Ca^2+^-entry through the PM NSCC forms a microdomain of high cytosolic Ca^2+^. If the cytosolic Ca^2+^-binding site in the vacuolar TPC1 is within this microdomain, the TPC1 opening is promoted. The “ignition” of TPC1 by high local cytosolic Ca^2+^ is facilitated by TPC1clustering, which in turn can amplify the initial cytosolic Ca^2+^ signal locally via vacuolar Ca^2+^ release. High cytosolic Ca^2+^ can activate several ion channels in the PM (not shown) and vacuolar K^+^ selective TPK channels. The activation of TPC1 channels is transient, and their inhibition by ROS from the vacuolar side can be a part of the feedback mechanism. **b** TPC1 and tonoplast excitability. Initially, a slightly cytosol negative transtonoplast electrical potential difference is set by electrogenic vacuolar H^+^ pumps (e.g V-ATPase) and a background activity of ion transporters. K^+^ release by TPK and TPC1 upon electrical stimulation generates vacuolar membrane depolarization (transtonoplast potential becomes more cytosol positive, approaching K^+^ equilibrium potential) with a stimulus strength-dependent duration. The shape of the voltage response, mediated by TPC1/TPK module, is shown above. This depolarization is rapidly transmitted to the whole membrane, changing the balance of the Ca^2+^/nH^+^ tonoplast antiport and reverting its operation mode to the net vacuolar Ca^2+^ release, providing *n* > 2. It has been demonstrated that TPK1 opening is somehow coupled to the activation of TPC1. In addition to a communication via local cytosolic Ca^2+^ changes as in **a**, we proposed a direct coupling of the TPK opening with the conformational changes of the TPC1 upon the activation of the latter. The advantage of this is that no full activation and opening of the TPC1 is required, but it can be an intermediate conformational state, sensed by TPK1, e.g. the one associated with the movement of the VSD2 by a depolarization stimulus (see Fig. 1b). In such a way, K^+^ flux will be through the TPK only, whereas TPC1 will be in a non-conducting pre-activated state, which can be reached already at resting cytosolic Ca^2+^. For this working hypothesis TPC1 and TPK must be in a close physical contact and their interaction can be realized via auxiliary proteins or even be a direct one (e.g. via interaction of their C-termini). See text for more details

We already mentioned that even at 2 μM cytosolic Ca^2+^, a maximal level for a bulk Ca^2+^ signal, the TPC1-mediated Ca^2+^ flux is rather small. But what about local cytosolic Ca^2+^ concentration? It is long known from experiments on animal cells that within 100 nm proximity of an open Ca^2+^-conducting channel local free Ca^2+^ can approach the level of up to 50 μM (Rizzutto and Pozzan 2006). Providing, such high Ca^2+^ microdomains can be generated in plant cells, at such a high local Ca^2+^ level the TPC1 activity becomes significant and the electrochemical gradient for Ca^2+^ is still cytosol-directed. Therefore, TPC1 channels in this case can locally amplify and/or sustain the initial Ca^2+^ signal, emitted by a partner Ca^2+^-permeable channel (Fig. 2a). This channel can be in a different membrane, e.g. endoplasmic reticulum or plasma (PM) one, which comes into a close contact with the tonoplast. Rather than generating a global Ca^2+^ release from the vacuole, which is fatal, in such arrangement the vacuole is split into many self-amplifying local Ca^2+^ circuits. For instance, GlR3.3 and 3.6 channels in the PM, which believed to be Ca^2+^-permeable, crosstalk with TPC1 during Ca^2+^ signaling associated with aphid feeding (Vincent et al. 2017). For the Ca^2+^ wave along the root induced by NaCl the partner channel is that one which mediates the ROS-activated Ca^2+^ influx. This functional circuit requires also Ca^2+^-dependent activation of the main ROS-producing enzyme of the plasma membrane, NADPH-oxidase or NOX (Evans et al. 2016). In case of the TPC1, ROS acts as a negative regulator via a soluble vacuolar factor (Pottosin et al. 2009). This mechanism can explain the cessation of the ROS and Ca^2+^ signal at the backside of the wave. Such feedback is also essential to prevent a simultaneous operation of many self-amplifying Ca^2+^ release circuits and detrimental global vacuolar Ca^2+^ release. The crosstalk between a plasma membrane Ca^2+^ influx channel and TPC1 can be indirectly evidenced by the fact that the loss-of-function TPC1 mutant was unable to close stomata specifically in a response to high external Ca^2+^ and that the principle hyperpolarization activated Ca^2+^ influx, which is involved in stomata closure, shares many similarities with the ROS-activated Ca^2+^ current (Pei et al. 2000; Islam et al. 2010). On the other hand, partner channel can be also another TPC1, providing the two TPC1 are colocalized in a proximity. Evans and co-workers (2016) analyzed the mechanism of the salt induced Ca^2+^ wave propagation along the root, applying a “bushfire” mathematical model. By a comparison of the observed frequency to find *n* TPC1 channels in a small membrane patch with the prediction by the Poisson distribution they concluded that the TPC1 channels are clustered. According to their theoretical considerations the clustering slows down the Ca^2+^ wave because the increased source release intensity of such a cluster does not compensate for increased distance between Ca^2+^ release sources. However, Evans and co-workers considered that the individual TPC1 channels in a cluster do not interact, so that the release strength in a cluster of *n* channels is just increased by a factor of *n*. Analysis of the surface density of active TPC1 channels suggests that the average distance between the neighboring channels is about 1 μm (Schulz-Lessdorf and Hedrich 1995; Pérez et al. 2008). This is too large to create the high Ca^2+^ microdomain. But it can be created if the TPC1 channels are clustered (Fig. 2a). We have experimentally demonstrated that the openings of several individual TPC1 channels in a small isolated patch are dependent one from another (Pottosin and Dobrovinskaya 2016). This argued for a short distance communication, either via Ca^2+^ flux or via a direct physical interaction. Notably, a dimerization of the TPC1 channels via C-termini was observed, and its disruption resulted in silent channels, not responding to cytosolic Ca^2+^ and voltage (Larisch et al., 2016).

#### TPC1 channels function may not require vacuolar Ca^2+^ release: vacuolar excitability

Electrical excitability was recently demonstrated for a higher plant tonoplast, which required a concerted function of the TPC1 and K^+^-selective TPK1 vacuolar channels (Jaślan et al. 2019). These experiments have been performed at zero vacuolar Ca^2+^, which not just decreased a threshold for the TPC1 activation, but also excluded from the consideration any mechanism, relied on the vacuolar Ca^2+^ release. Because K^+^ conductance of the TPC1 exceeds that of Ca^2+^ by more than order of magnitude, the direct contribution of the latter in the electrogenesis by TPC1 can be neglected. Tonoplast excitability was manifested by a prolonged depolarization (post-stimulus voltage plateau), whose duration was strongly dependent on the stimulus strength, reaching few seconds at current injections in the range of hundred pA. Plateau disappeared in lack-of-function TPC1 mutants and upon introduction of physiological (1 mM) vacuolar Ca^2+^. The role of K^+^ release via K^+^-selective tonoplast channels was proven by a substantial decrease of the post-stimulus plateau in TPK knock-out mutants. Whereas TPC1 is readily permeable for Cs^+^, with a conductance of about half of that for K^+^ (Pottosin and Dobrovinskaya 2014), Cs^+^ blocks TPK channels and substitution of K^+^ for Cs^+^ at the vacuolar side abolished the post-stimulus plateau (Jaślan et al. 2019). Tonoplast electrical potential difference can be clamped in vivo and was associated with a cytosolic Ca^2+^ release or uptake in case of de- or hyperpolarization, respectively (Dindas et al., 2021). In this range of potentials, a Ca^2+^-permeable channel can only mediate vacuolar Ca^2+^ release, whereas vacuolar Ca^2+^-ATPase only vacuolar Ca^2+^ uptake. To account for experimentally observed dependence of Ca^2+^ flux, when it switched from Ca^2+^ uptake to Ca^2+^ release upon a depolarization within a subzero voltage range the operation of an electrogenic Ca^2+^/nH^+^ antiporter with *n* > 2 has to be postulated (Dindas et al., 2021). Thus, it has been concluded that such a depolarization generates a cytosolic Ca^2+^ signal via an imbalance, which reverts the direction of the Ca^2+^/nH^+^ antiport and not via a direct Ca^2+^ release by TPC1 channels (Fig. 2b). It should be noted that the exact mechanism maybe more complex and involve more transport components, because at *n* = 3 and higher the reversal potential of a Ca^2+^/nH^+^ antiporter becomes rather positive at cytosol/vacuole ΔpH = 2, which is close to physiological pH difference. Theoretical thermodynamic predictions for a Ca^2+^/nH^+^ antiporter only fit experimental observations for the ΔpH range between 1 and 1.5, which is an underestimate. Of note also is that the function of the TPC1/TPK module in vivo has not been demonstrated directly but mimicked by externally applied voltage stimuli of different strength and duration. As mentioned above, with 1 mM vacuolar Ca^2+^, which is close to the in vivo situation, the operation of this module was abolished. Nevertheless, the above mechanism of the generation of Ca^2+^ rise, which does not require a channel-mediated vacuolar Ca^2+^ release but only a channel-mediated tonoplast depolarization via K^+^ release, is an intriguing possibility. Electrical signal propagates rapidly, thus it can be rapidly transduced to the cytosolic Ca^2+^ signal at any intracellular location, which in turn can be passed to a neighboring cell via plasmodesmata or be communicated to the PM ion channels via a calcineurin B-like protein (CBL)/CBL-interacting protein kinase cascade (Dindas et al., 2021).

#### A mechanism independent on ion conductance of the TPC1

When modeling the plateau duration on the stimulus strength a very unexpected theoretical result was obtained: the activity of the TPK channels has to follow/ to be coupled with a voltage-dependent gating of the TPC1 ones, otherwise the simulated post-stimulus depolarization duration became independent on the stimulus strength, in contrast to experimental observations (Jaślan et al. 2019). The coupling factor in this case cannot be Ca^2+^, released by TPC1 from the vacuole, because this ion was omitted from the experimental solutions. Therefore, we think in a different coupling mechanism. In the last two decades there is an accumulated evidence for a non-canonic (independent on ion conductance) function of members of voltage-dependent channels superfamily, including various Shaker K^+^ (K_v_), Na_v_ and Ca_v_ channels (Kaczmarek 2006; Arcangeli and Becchetti 2010; Lee et al., 2014). Respective signaling processes were mediated by channel protein interactions with auxiliary and signaling, as well as scaffolding proteins, elements of cytoskeleton and β-integrins, so that channels form a part of a signalosome complex. Different voltage-dependent channels can also directly interact each with other, as it was demonstrated for large conductance Ca^2+^-activated K^+^ and Ca_v_ channels (Berkefeld et al. 2006), whereas some K_v_ and Ca_v_ channels functionally interact via auxiliary proteins (Anderson et al. 2010). A rather interesting example is the case of the voltage-dependent Kv1.3 channel, which can perform the signaling function without K^+^ conductance (a poreless mutant) whereas mutations, increasing the voltage activation threshold, were inefficient. It has been concluded that the cytosolic signaling domain, part of which is formed by certain scaffold protein, senses the conformational changes, associated with the VSD movement in Kv1.3 (Cidad et al. 2012, 2021). Thus, we propose that TPC1 and TPK proteins can functionally interact, either directly or via auxiliary proteins, and that TPC1 acts as an external voltage sensor for the TPK (Fig. 2b). This assumption is sheer hypothetical at the moment, but it can be verified experimentally, providing a poreless mutant of the TPC1 is generated. The attractive side of this hypothetical model is obvious: as far as no TPC1 opening is required and intermediate conformational states, associated with the VSD movements (Fig. 1b), can be already sensed via protein-protein interaction, this reduces the threshold for the activation of this signaling pathway as compared to a threshold for the complete TPC1 channel activation/opening. In particular, no increase of cytosolic Ca^2+^ should be required anymore.

## Conclusions

There is a solid body of evidence for functional roles of the TPC1 channels in stress signaling (Table 1). In the last years a rather detailed information was obtained on the structural rearrangements and key involved residues, which underlie the TPC1 gating by cytosolic and vacuolar Ca^2+^ and voltage (Ye et al. 2021; Dickinson et al. 2022). Taken together with existing advanced kinetic modeling, we can quantitatively predict the observed channel activity at any set of Ca^2+^ and voltage conditions. This prediction works fine for resting conditions and explains the experimentally observed low channel probability. Yet, there is a gap in understanding how the TPC1 works upon signaling. Here we considered possible mechanisms of the TPC1 integration into Ca^2+^, ROS and electrical signaling processes. To overcome the high threshold for the TPC1 activation we assume the generation of high Ca^2+^ microdomains, or, alternatively, a direct sensing of the TPC1 conformational changes, which occur before cytosolic Ca^2+^ binding and channel opening. In both cases, the integration of the TPC1 into the long-distance signaling is paradoxically relied on nanolocal interactions. We have critically discussed different and non-exclusive modes of the TPC1 integration, which are dependent on its Ca^2+^ or K^+^ conductance, or ion flux independent voltage sensing. To address these issues experimentally, specific channel mutations should be tested, which include those affecting voltage gating or pore conductance and selectivity. Some relevant mutants, such as for instance a Na^+^ vs Ca^2+^selective one, already exist (Guo et al. 2017). The target residues, whose mutation can eventually lead to the TPC1 with a preferential Na^+^ and K^+^ vs Ca^2+^ permeability, are pinpointed (Navarro-Retamal et al. 2021). Some mutants, with increased voltage activation threshold are available (Guo et al. 2016; Jaślan et al. 2016) and further mutants with altered voltage-gating can be obtained on demand. A crucial step will be the generation and testing of a poreless TPC1 mutant with an intact voltage gating.

## Data Availability

Not applicable.

## Abbreviations

Ca_v_: K_v_ or Na_v_, voltage dependent Ca^2+^, K^+^ or Na^+^ channels
CBL: Calcineurin B-like protein
cryo-EM: Cryogenic electron microscopy
E_Ca_: Equilibrium potential for Ca^2+^
EF-hand: Canonical Ca^2+^ binding motif
GlR: Glutamate receptor channel
K_d_: Dissociation constant
MIFE: Noninvasive microelectrode ion flux estimate technique
NOX: NADPH oxidase
NSCC: Nonselective cation channel
PA: Polyamine
PD: Pore domain
PM: Plasma membrane
Put: Putrescine
ROS: Reactive oxygen species
SF: Selectivity filter
TPC1: Two-pore cation channel 1
TPK: Tandem Pore K^+^ channel
TM or S1–12: Transmembrane segment
Spm: Spermine
VSD: Voltage sensing domain
